# Extracellular Adenosine Contributes to the Hydrogen Peroxide-Induced Calcification of Cultured Tendon Cells

**DOI:** 10.3390/cimb48030244

**Published:** 2026-02-26

**Authors:** Tomomi Sakuma, Chantida P. N. Mahasarakham, Xin Lin, Hiroyuki Yoshitake, Akira Nifuji, Masaki Noda, Yoichi Ezura

**Affiliations:** 1 Department of Maxillofacial Surgery, Institute of Science Tokyo (IST), Tokyo 113-8510, Japan; 2 Frontier Research Unit, Skeletal Molecular Pharmacology, Medical Research Institute, Tokyo Medical and Dental University (TMDU), Tokyo 113-8510, Japan; 3 Department of Restorative Dentistry, Faculty of Dentistry, Khon Kaen University, Mitrparb Rd., Khon Kaen 40002, Thailand; 4 Department of Pharmacology, Tsurumi University School of Dental Medicine, Tsurumi 230-8501, Japan; 5 Center for Stem Cell and Regenerative Medicine, Institute of Science Tokyo (IST), Tokyo 113-8510, Japan; 6 Department of Joint Surgery and Sports Medicine, Institute of Science Tokyo (IST), Tokyo 113-8510, Japan; 7 Faculty of Occupational Therapy, Teikyo Heisei University, Tokyo 170-8445, Japan

**Keywords:** heterotopic ossification, tendon cells, adenosine, extracellular nucleotides

## Abstract

Background: Well-known risk factors for soft tissue heterotopic ossification (HO) include aging and mechanical stress, which may be linked to oxidative stress and downstream nucleotide metabolites. Thus, we investigated the involvement of extracellular ATP (ex-ATP) and its metabolites in the oxidative stress-induced mineralization of TT-D6 cells and primary mouse tendon cells. Methods: An osteogenic culture with the intermittent addition of hydrogen peroxide was monitored for two weeks using metabolomic and gene expression analyses. Results: Calcium deposition was significantly enhanced by 0.3 mM hydrogen peroxide in the osteogenic media after 2 weeks, with minimal calcification in its absence. Similar results were observed in a medium transfer experiment using 3-day-old hydrogen peroxide-treated conditioned medium, which led to an increased expression of osterix and alkaline phosphatase. Metabolomic analysis revealed a gradual increase in ex-ATP and its metabolites, including ADP, AMP, and adenosine, in the medium. The metabolite increase was enhanced by hydrogen peroxide after 12 h. Moreover, exogenous adenosine (100 μM) increased mineralization in osteogenic media. Additionally, 1 μM dipyridamole, an inhibitor of equilibrative nucleoside transporter 1 (Ent1), also increased it in response to low-dose (0.1 mM) hydrogen peroxide. Conclusions: The enhanced osteogenic calcification of the tendon cell culture by hydrogen peroxide was associated with an increase in extracellular nucleotide metabolites, especially adenosine, with some evidence of causality.

## 1. Introduction

Heterotopic ossification (HO) occurs in human soft tissues, including the skin, brain, blood vessels, skeletal muscle, and tendons, in certain pathological conditions [1,2,3]. In the context of vascular and other connective tissue ossification, aging is often the primary risk factor for HO, and its effects are mediated by the senescence-associated secretory phenotype, which is linked to inflammation and oxidative stress. Another well-known risk factor for HO is the occurrence of injuries, burns, and surgery [2,3,4], which also stresses cells by generating reactive oxygen species (ROS). Culture experiments have repeatedly demonstrated that moderate oxidative stress induced by hydrogen peroxide can strengthen mesenchymal cell mineralization [5,6,7]. However, the molecular mechanism of this induction remains to be elucidated.

In the pathogenesis of traumatic HO, an involvement of extracellular ATP (ex-ATP) has also been suggested [8]. Because oxidatively stressed or dying cells release ATP into the extracellular space through several mechanisms, including the opening of hemichannels on the plasma membrane [9,10], we assumed that oxidative stress is causally related to ATP release. Increased extracellular ATP (ex-ATP) mediates cellular signaling through transmembrane purinergic receptors belonging to the P2X and P2Y families [10,11,12], and such signaling has been reported to increase osteogenesis of mesenchymal cells. However, the literature reports somewhat controversial findings regarding osteogenic responses. We assume that this complexity may be related to the multiple occurrences of nucleotide receptors and the overlapping binding of each receptor. That is, ex-ATP and its metabolites, ADP, and AMP can bind to many P2 receptors [10,11], and adenosine binds to P1 receptors A1 to A3 [8]. Thus, a complex interplay among multiple signaling molecules, which fluctuate with the context and cell type, may explain the results of different culture experiments [10,11,12,13]. Nevertheless, we can hypothesize that one or two metabolites are critical determinants of the final mineralization.

Molecular and genetic insights into congenital disorders associated with HO have implicated a complex involvement of ex-ATP in HO. The importance of ex-ATP was initially indicated by causative mutations found in patients with “generalized arterial calcification infancy” (GACI; OMIM #208000) in the ectonucleotide pyrophosphatase/phosphodiesterase 1 (*ENPP1*) gene, which encodes one of the extracellular hydrolyzing enzymes of ex-ATP [14,15]. Early studies suggested a mechanism involving a balanced modulation between its byproducts, Pi, and PPi [14,15]. However, subsequent studies identified loss-of-function mutations in ATP-binding cassette subfamily C member 6 (ABCC6) in a small number of GACI cases (GACI2; OMIM #614473) [16]. These findings, which revealed that pathologies resembling GACI1 can develop even in the absence of ENPP1 dysfunction, reaffirm that reduced PPi alone does not fully explain the pathogenesis of this disease.

This notion that ex-ATP itself is essential led us to further consider the involvement of ex-ATP’s hydrolysis products, AMP and adenosine, because a loss-of-function mutation in the 5′ nucleotidase ecto (*NT5E*) was found in “hereditary arterial and articular multiple calcification syndrome” [17]. The findings of a defective adenosine transporter gene, specifically equilibrative nucleoside transporter 1 (*ENT1*; also known as *SLC29A1*), supported the involvement of adenosine in connective tissue HO in humans and mice [18,19]. In addition, transcriptomic analysis of mesenchymal cells in an osteogenic culture suggested the involvement of its receptors [8]. However, a paradox arises: HO induced by *NT5E* mutation may suggest an inhibitory effect of adenosine on calcification, whereas HO induced by *ENT1* mutation may suggest an accelerating effect of adenosine if extracellular ATP is involved. To reconcile this controversial evidence, we need to understand how extracellular ATP, AMP, and adenosine levels may change after oxidative stress in mesenchymal cells.

Therefore, in this study, we investigated the possible link between oxidative stress and extracellular nucleotide metabolites in cultured tendon cells by adding hydrogen peroxide to the osteogenic culture medium. We first used primary tendon cells obtained from the plantar region of the flexor digitorum longus (FDL) tendon in adolescent mice, which have been utilized in histological tendon studies [20]. In addition, the TT-D6 cell line [21], established in our laboratory in 2003 from the Achilles tendons of transgenic mice expressing the SV40 T antigen, was used. Using these two systems, we examined the effects of adenosine, an ex-ATP metabolite, on increased mineralization in tendon cell culture experiments.

## 2. Materials and Methods

### 2.1. Animals

Inbred C57Bl6/J mice aged 6 to 8 weeks were purchased from a vendor (Clea-Japan, Inc., Tokyo, Japan) and euthanized using an overdose of isoflurane and cervical dislocation to prepare primary FDL tendon cells. The experiments were conducted in accordance with the protocol, which complied with ARRIVE guidelines, and were approved by the Animal Welfare Committee of Tokyo Medical and Dental University (Approval Numbers: A2017-082A, A2018-180A, and A2019-222A).

### 2.2. Cell Culture

The FDL tendons obtained from the euthanized mice were minced with a scalpel after removing vascular and muscular tissues. The minced tendons were sequentially digested 5 times for 30 min with collagenase (1 mg/mL) and dispase II (2 mg/mL) in alpha-MEM at 37 °C. The homogenized cell suspension was filtered through a 40 μm cell strainer (BD Biosciences, San Jose, CA, USA) and seeded into a 60 cm^2^ culture dish. It was then cultured in a humidified chamber at 37 °C in 5% CO_2_. The culture medium was changed on days 1 and 4, and the cells were reseeded on day 7 (first passage; P1). The cells were expanded until passage 2 and then plated in multi-well plates at around 2–5 × 10^3^ cells/cm^2^ in alpha-MEM supplemented with 10% FBS and an antibiotic–antimycotic mixture (Thermo-Fisher Scientific, Waltham, MA, USA: Cat.#15240062).

The established tendon cell line, TT-D6, was cultured in alpha-MEM supplemented with 0.5–10% FBS at 33 °C as previously described [21]. However, for differentiation assays, the cells were maintained at 37 °C and passaged to be plated at 5–20 × 10^3^ cells/cm^2^ in multi-well plates. Osteogenic induction was performed by beta-glycerophosphate (10 mM) and ascorbic acid (50 μM) with and without the addition of hydrogen peroxide or other reagents. Based on the results of preliminary experiments (Supplementary Figure S3), this study used hydrogen peroxide concentrations of 0.1–0.3 mM, selecting 0.3 mM for most experiments.

Conditioned medium (CM) was collected during medium changes on days 3, 6, 9, and 12 from wells treated with 0.3 mM hydrogen peroxide three days prior. Each time, half the volume of the CM was applied to the neighboring wells on the same culture plate after the same volume of fresh osteogenic medium without hydrogen peroxide had already been added.

### 2.3. Alizarin Red Staining

Mineralization status was evaluated by performing alizarin red staining as previously described [22] on days 6, 9, 12, and 15 on the culture plates. Briefly, the cells were fixed with 4% paraformaldehyde for 5 min and washed with deionized water. Alizarin red S solution (0.5%, pH = 6.4) was added to the cells for about 1 min and then washed with clean water. Photographic images of the stained wells were evaluated densitometrically using ImageJ 1.44p (National Institutes of Health, Bethesda, MD, USA). In some experiments, the alizarin red was eluted in the solubilizing solution (20% methanol and 10% acetic acid) for 30 min at room temperature, and the solution was quantified by measuring the optical density at 450/650 nm using a microplate reader, “infiniteF50R” (Tecan, Männedorf, Switzerland).

### 2.4. Gene Expression Analysis

The total RNA was isolated from the cultured cells using TRIzol reagent (Thermo-Fisher Scientific, Waltham, MA, USA) according to the manufacturer’s protocol. Reverse transcription (RT) was performed using a High-Capacity cDNA Reverse Transcription Kit (Thermo-Fisher Scientific) with 1 μg total RNA. Quantitative real-time PCR (qPCR) was performed using a StepOne instrument (Applied Biosystems; Thermo Fisher Scientific, Waltham, MA, USA). Aliquots of 10 μL reagent mixture containing 2 μL of cDNA samples, 0.2 μM forward and reverse primers, and 5 μL of FAST SYBR^®^ Green Master Mix (Applied Biosystems) were applied and analyzed. The data were normalized by the levels of the glyceraldehyde-3-phosphate dehydrogenase (*Gapdh*) gene, which were confirmed to be stable in our culture conditions in TT-D6 cells (Supplemental Figure S1). The primer sequences for these target genes are provided in Table 1.

### 2.5. Metabolomic Analysis of Nucleotides and Nucleosides

The CM of the TT-D6 cells was sampled at various time points (0–1000 min after medium change) on the first day of the mineralization culture with or without 0.3 mM hydrogen peroxide treatment. The samples were frozen immediately and stored until they were transferred to the company’s laboratory (Infiniti-Lab. Tokyo, Japan), where metabolite quantification was carried out using an Agilent 1290 Infinity liquid chromatography (LC) system (Agilent Technologies, Santa Clara, CA, USA) coupled to an Agilent 6470 triple quadrupole mass spectrometer, equipped with an Agilent Jet Stream source operating in electrospray ionization (ESI) mode. Specifically, a 200 µL aliquot of culture medium was mixed thoroughly with 200 µL of acetonitrile containing the internal standard (20 µM methionine sulfone), and 100 µL of the centrifugal product was filtered by a Millipore 5 kDa molecular weight cutoff filter (9100× *g* and 4 °C for 180 min) [23,24] and subjected to mass spectrometry (LC-MS/MS) analysis.

Chromatographic separation was performed on an InfinityLab Poroshell 120 HILIC-Z column (2.7 µm, 2.1 mm × 100 mm i.d., Agilent) maintained at 45 °C. The mobile phase for nucleotides consisted of solvent A (HPLC-grade water containing 10 mM ammonium acetate and 5 µM medronic acid) and solvent B (80% acetonitrile containing 10 mM ammonium acetate and 5 µM medronic acid). Separation was achieved using a linear gradient of solvent B applied with an Infinity II binary pump, starting at 100% B and reducing to 90% over 0.5 min, followed by a gradient from 90% to 70% over 9.5 min. The column was then re-equilibrated at 100% B for 3 min, resulting in a total run time of 13 min per sample. The flow rate was maintained at 0.3 mL/min, and the injection volume was 3 µL [25]. Similarly, nucleosides were separated with distinct solvent A’ (HPLC-grade water containing 20 mM ammonium acetate) and solvent B’ (acetonitrile). A linear gradient of solvent B’ started at 95% B and was held at 95% for 1 min, followed by a gradient from 95% to 69% over 4 min, and then from 69% to 50% over 1 min, with 50% B’ held constant for an additional 1 min. The column was re-equilibrated at 95% B for 6 min, resulting in a total run time of 13 min per sample. The flow rate was set at 0.3 mL/min, and the injection volume was 1 µL [26]. Mass spectrometric analysis was performed in positive ESI mode with multiple reaction monitoring (MRM), using unit resolution for both the Q1 and Q3 quadrupoles. The MRM transitions for the nucleotides and nucleosides are detailed in Supplemental Tables S1 and S2, respectively. The ESI source parameters were optimized as follows: For nucleotides: gas temperature, 200 °C; gas flow rate, 10 L/min; nebulizer pressure, 40 psi; sheath gas temperature, 300 °C; sheath gas flow rate, 12 L/min; and capillary voltage, 3500 V. All measurements were conducted in positive ionization mode. For nucleosides: gas temperature, 200 °C; gas flow rate, 10 L/min; nebulizer pressure, 40 psi; sheath gas temperature, 300 °C; sheath gas flow rate, 12 L/min; and capillary voltage, 3500 V. Data acquisition was performed in positive and negative ionization switching mode. Data analysis was conducted using MassHunter Quantitative Analysis software (version 10.1.733.0). Nucleotides and nucleosides were quantified using the internal standard method. Calibration curves were constructed using standard solutions containing analytes at concentrations reflective of those expected in the culture medium. Each curve consisted of 15 calibration points, spanning concentrations from 1 nM to 50 μM.

### 2.6. Statistical Analysis

Statistical analysis was performed on the data obtained by cell biological and histological measurements. When applicable, the Kolmogorov–Smirnov (K-S) test was used to assess normality, and the data are presented as mean ± standard deviation (mean ± SD). Student’s *t*-test and one-way or two-way analysis of variance (ANOVA) were applied when the normal distribution was supported. Student’s *t*-test with Bonferroni’s approximation or Dunnett’s method was applied as a post hoc test for ANOVA.

## 3. Results

### 3.1. Hydrogen Peroxide Induces the Calcification of Cultured Tendon Cells (TCs)

**Figure 1:**
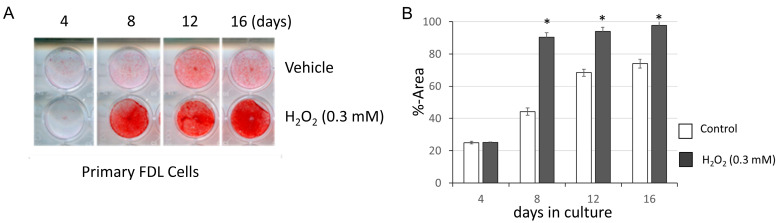
Hydrogen peroxide enhances primary tendon cell mineralization if added to the osteogenic medium. (**A**) Primary tendon cells from mouse FDL tendons were cultured in a standard osteogenic medium with or without addition of 0.3 mM of hydrogen peroxide for 16 days, and mineralization was evaluated by alizarin red staining. (**B**) The staining was quantified by densitometric analysis. * *p*-value < 0.05, determined by Student’s *t*-test with Bonferroni’s approximation as post hoc test of 2-way ANOVA.

We first examined whether hydrogen peroxide enhances the mineralization of FDL tendon cells from adolescent mice (6–10 weeks of age) in an osteogenic induction culture. By applying different concentrations of hydrogen peroxide, we found that tendon cell mineralization was significantly enhanced at lower concentrations (<0.3 mM; Figure 1). Higher concentrations of hydrogen peroxide tended to induce cell death (Supplemental Figure S3). However, low concentrations of hydrogen peroxide, below 0.3 mM, were acceptable for continuing the osteogenic assay with lower levels of cell death.

**Figure 2:**
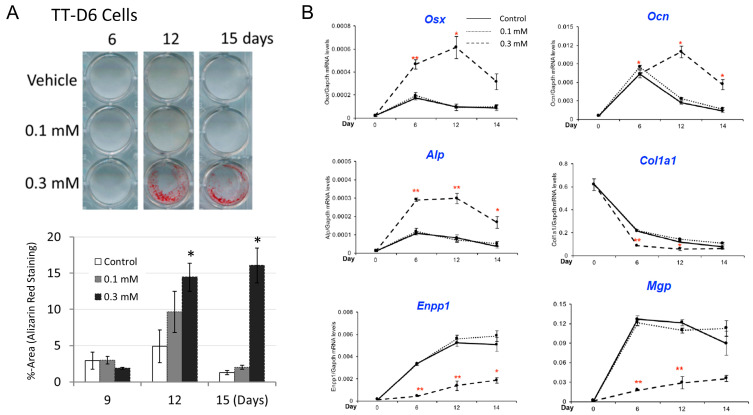
Hydrogen peroxide enhanced tendon cell mineralization when added to the osteogenic medium. (**A**) TT-D6 cells were cultured in a standard osteogenic medium with or without addition 0.1 or 0.3 mM of hydrogen peroxide for 16 days, and mineralization was evaluated by alizarin red staining (**upper panel**). The staining was quantified by densitometric analysis (**bottom panel**). Each experimental condition consisted of 3 wells in parallel, and Dunnett’s test was used as a post hoc test for the ANOVA. (**B**) Quantitative RT-PCR analysis was performed for the culture experiment in panel A. Osx, osterix; Ocn, osteocalcin; Alp, tissue nonspecific alkaline phosphatase; Col1a1, type I collagen alpha 1 chain; Enpp1, ectonucleotide pyrophosphatase/phosphodiesterase 1; Mgp, matrix Gla-protein. * *p*-value < 0.05, ** <0.01, determined by Student’s *t*-test with Bonferroni’s approximation as post hoc test of 2-way ANOVA.

Because the culture conditions for primary tendon cells vary across preparations, we conducted additional experiments using the precursor tendon cell line TT-D6 [20], which is basically resistant to inductive mineralization in standard osteogenic media. The addition of 0.3 mM of hydrogen peroxide to a standard osteogenic medium resulted in significant mineralization after 12–15 days of culture, as evaluated by alizarin red staining, but the addition of 0.1 mM was not enough (Figure 2A). The increased mineralization was associated with an increased expression of osterix (*Osx*), osteocalcin (*Ocn*), and tissue nonspecific alkaline phosphatase (*Alp*) (Figure 2B), consistent with the previous results in vascular cells. In addition, the expression of endogenous mineralization inhibitors, *Enpp1* and matrix Gla-protein (*Mgp*), decreased during the culture period, suggesting a potential influence on the hydrogen peroxide-induced enhancement of mineralization. On the other hand, the expression of another mineralization inhibitor, *Ent1* (also known as *Slc29a1*), which is an adenosine transporter, was not significantly altered.

### 3.2. Hydrogen Peroxide Induces TC Calcification Through Secondary Secreted Factors

**Figure 3:**
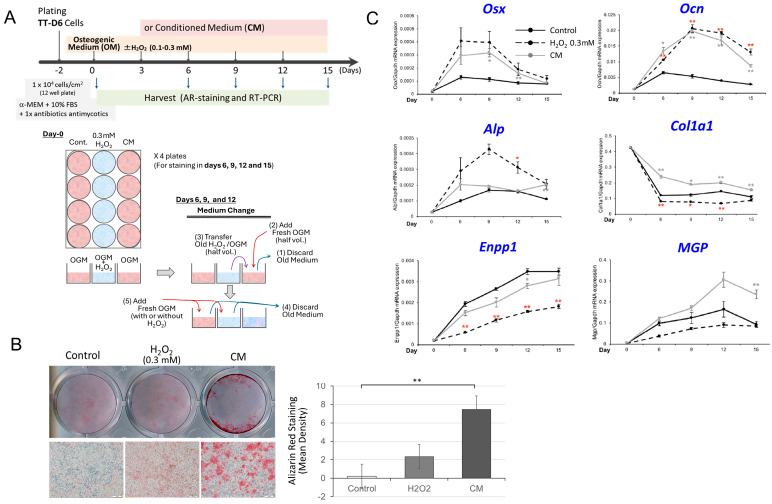
Transferring a 3-day-old conditioned medium treated with hydrogen peroxide to fresh osteogenic medium enhances TT-D6 cell mineralization. (**A**) A schematic diagram of the experimental protocol is shown in the **upper panel**, and the CM collection and application are explained in the **lower panel**. OGM: osteogenic culture medium. (**B**) Representative image of the wells on day 15 with alizarin red staining. Densitometric analysis of the staining (*n* = 3) was performed to semi-quantify the results (statistically non-significant). (**C**) Quantitative RT-PCR analysis of this experiment. Each experimental setting had 3 wells. Similar results were obtained from three independent experiments. * *p*-value < 0.05, ** <0.01, determined by Student’s *t*-test with Bonferroni’s approximation as a post hoc test of 2-way ANOVA.

Since we hypothesized that the increased mineralization in the above experiments may depend on the secretory factors secondary to oxidative stress in the tendon cells, at least in part, the following experiments were conducted using the conditioned medium (CM) of TT-D6 cells, where 0.3 mM hydrogen peroxide was added to the osteogenic medium three days prior. During each medium change on days 3, 6, 9, and 12, fresh CM was transferred to the adjacent wells in parallel cultures, without hydrogen peroxide treatment, in a 1:1 ratio. Then, we observed increased mineralization in these assays on day 15 or later (Figure 3A,B). The enhanced calcification was associated with an increased expression of *Alp* and *Ocn* (Figure 3C), as observed in the experiments presented in Figure 2. However, since the expression of the *Enpp1* or *Mgp* gene was not suppressed, the positive effect on calcification cannot be solely responsible for such responses in hydrogen peroxide-treated cells.

As noted above, even at low doses, hydrogen peroxide loading can induce cell death; however, this was not observed in the cells treated with CM using the above-described method. Therefore, the accelerated mineralization was not due to residual hydrogen peroxide or the dying cells but to secretory factors induced by hydrogen peroxide.

### 3.3. Hydrogen Peroxide Induces the Secretion of Nucleotide Metabolites from Tendon Cells

**Figure 4:**
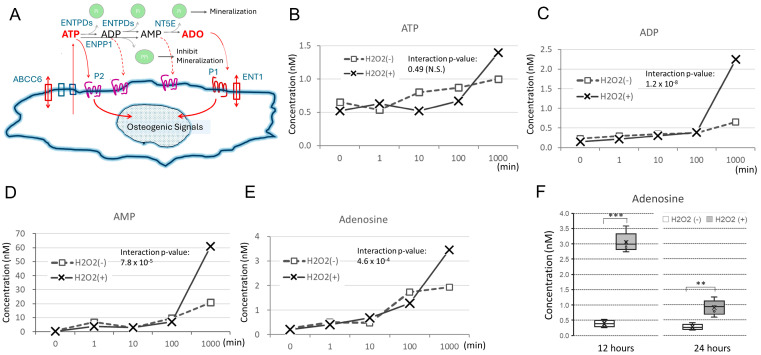
The metabolites of ex-ATP increased in the culture medium by 1000-fold (16.7 h) during osteogenic induction. Metabolomic analyses were performed using liquid chromatography and mass spectrometry on the conditioned medium from TT-D6 cells during osteogenic induction with or without hydrogen peroxide treatment (0.3 mM). (**A**) The hydrolysis pathway of ex-ATP and cellular responses to the metabolites are schematically presented. (**B**–**E**) Time series analysis of nucleotides and nucleosides over 0, 1, 10, 100, and 1000 min was performed with duplicate samples. The horizontal axis is the time, and the vertical axis is the concentration. The data plot represents the mean duplicate values (*n* = 2). Statistical analysis was conducted using a 2-way ANOVA, and the interaction *p*-value was evaluated. (**F**) Adenosine levels in the osteogenic medium with or without hydrogen peroxide treatment (0.3 mM) were statistically analyzed at 12 and 24 h. The number of wells in each setting was 5 (*n* = 5). N.S. not significant, *** *p* < 1.0 × 10^−5^, ** *p* < 0.001, determined by Student’s *t*-test.

Since ex-ATP and its metabolites can influence the mineralization of cultured cells in various ways [9,10], we investigated whether the levels of such metabolites increase after hydrogen peroxide treatment in an osteogenic culture of TT-D6 cells using LC-MS/MS (Figure 4). Metabolomic analysis revealed that ex-ATP levels were initially low at about 0.5 nM in the CM but increased to about 1.0–1.5 nM by 1000 min (corresponding to about 16 h) in both groups. However, a differential increase between the control and experimental groups compared with the hydrogen peroxide group was not evident (Figure 4B). In contrast, a significant rise in ADP and AMP was observed only in the group treated with hydrogen peroxide from 100 to 1000 min (Figure 4C,D). The gradual increase in adenosine levels was further enhanced by hydrogen peroxide during the same period (Figure 4E). Based on these observations, we formulated the following hypothesis. The rapid increase in extracellular ADP and AMP suggests that a large amount of ATP was hydrolyzed. The absence of a similar rise in ex-ATP levels during this period is reconcilable if we consider rapid internalization via the ABCC6 transporter in parallel. Similarly, only a moderate increase in adenosine compared to ADP and AMP (Figure 4C,D) may imply prompt cellular uptake of adenosine via the transporter Ent1. To test whether adenosine levels after 12 to 24 h of hydrogen peroxide treatment are higher than those in conditioned medium not treated with hydrogen peroxide, subsequent experiments (*n* = 5) were conducted. A significant increase in adenosine was confirmed at 12 h (*p* = 1.1 × 10^−7^ by Student’s *t*-test; *n* = 5), and this increase slightly reduced by 24 h (6.2 × 10^−4^) (Figure 4F). These observations suggest that the increased ADP, AMP, and subsequent adenosine may contribute to the enhancement of mineralization after hydrogen peroxide treatment rather than the increase in ex-ATP.

### 3.4. Adenosine Strengthens the Tendon Cell Mineralization Induced by Hydrogen Peroxide

**Figure 5:**
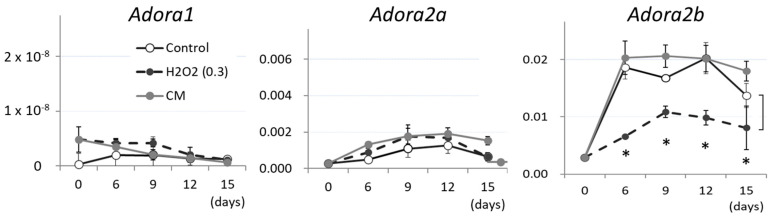
Expression of adenosine receptors during osteogenic induction culture of TT-D6. Expression levels of adenosine receptor type 1 gene (*Adora1*) and adenosine receptor type 2 genes (*Adora2a* and *Adora2b*) were analyzed by RT-PCR during osteogenic induction of TT-D6 cells with or without hydrogen peroxide treatment (0.3 mM). The vertical axis shows the ratio of the estimated number of molecules to that of Gapdh. * *p*-value < 0.05, determined by Student’s *t*-test with Bonferroni’s approximation as a post hoc test of 2-way ANOVA.

However, given the functional roles of genetic mutations in congenital disorders, as described in [8,17,18,19], a paradox remains regarding the presumed influence of extracellular adenosine. Assuming the influence of the differential expression of three adenosine receptor types [12], among which types 1 and 3 transmit inhibitory signals but type 2 transmits positive signals, we examined the expression of adenosine receptor types in TT-D6 cells by qRT-PCR. We found that the expression levels of type 2b and 2a were the highest and the second highest, while those of the other types were below detectable limits during the induction of osteogenic calcification in TT-D6 cells (Figure 5). Although a significant decrease in type 2b receptor levels was observed after hydrogen peroxide treatment (dotted lines in the right-most panel of Figure 5 compared to black solid line with open circles), such a suppression was not observed in the CM group (gray line in the right-most panel of Figure 5). Therefore, the decline in *Ador2b* expression induced by hydrogen peroxide was assumed not to contribute to the ex-ATP pathway that leads to enhanced TT-D6 calcification. This assumption supports the idea that the levels of the ligand adenosine should influence its net signal.

**Figure 6:**
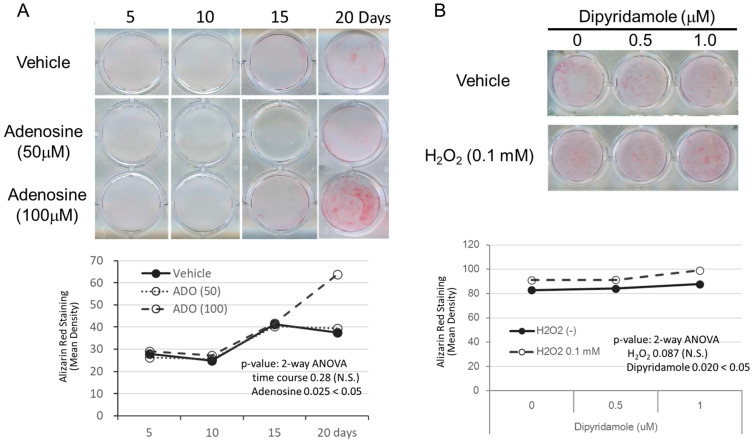
Adenosine or dipyridamole treatment enhanced mineralization by inducing osteogenic differentiation in TT-D6 cells cultured with a low concentration of hydrogen peroxide. (**A**) Osteogenic induction of TT-D6 cells was performed with or without extrinsic adenosine treatment at two concentrations (50 and 100 micromolar). (**B**) Osteogenic induction of TT-D6 cells was performed with a low concentration of hydrogen peroxide (0.1 mM), and two different concentrations of dipyridamole (0.5 and 1.0 micromolar) were evaluated by alizarin red staining. N.S. not significant. Statistical analysis was conducted using 2-way ANOVA.

We thus tested whether adenosine in the culture medium influences TT-D6 cell mineralization by adding it to the osteogenic medium. However, at low adenosine concentrations (10–100 nM) corresponding to the mass spectrometry analysis data (as shown in Figure 4), no significant changes in calcification were observed. Nevertheless, we observed that a very high adenosine concentration (100 micromolar) tended to increase mineralization compared with the standard osteogenic medium (Figure 6A).

We hypothesized that one possible reason why our assay required high concentrations of adenosine is its internalization by Ent1, whose inhibitory function in soft tissue HO has been demonstrated in human and mouse genetic analyses [18,19]. Thus, we applied an Ent1 inhibitor, dipyridamole, to the TT-D6 osteogenic culture and found that dipyridamole did not strengthen the ossified nodules but did so slightly when a low concentration (0.1 mM) of hydrogen peroxide was added (Figure 6B). Similar findings were obtained when dipyridamole was applied to the culture medium with a high concentration of adenosine but without hydrogen peroxide (Figure S4).

Next, we examined whether similar findings could be obtained in the primary FDL tendon cell culture (Figure S5). However, similar results were not observed when adenosine was added at the same concentrations in these cells, possibly due to differences in dose requirements. Indeed, osteogenic induction was enhanced by a higher dose of adenosine (200 micromolar) when dipyridamole was added (Figure S6).

Although statistical analyses of the repeated experiments did not indicate a significant effect in each experiment, possibly due to small sample sizes, these observations supported the above-described idea that an increase in extracellular adenosine may be a critical factor in hydrogen peroxide-induced tendon cell calcification during osteogenic induction culture experiments.

## 4. Discussion

This study investigated the effect of hydrogen peroxide on tendon cell mineralization, focusing on the hypothetical contribution of the secreted ex-ATP and its metabolites. First, we demonstrated enhanced osteogenic mineralization in the progenitor tendon cell line TT-D6 and in primary tendon cells following periodic treatment with hydrogen peroxide (0.3 mM). This enhancement was suggested to depend on secretory factors, as CM treatment yielded similar findings. Since LC-MS/MS analysis revealed increased levels of the hydrolysis products of ex-ATP at around 12 h after hydrogen peroxide treatment, we conducted further investigations and demonstrated that one of the byproducts, adenosine, may be involved in enhancing the mineralization induced by hydrogen peroxide. This is the first report to demonstrate that the concentrations of ex-ATP and adenosine increase following hydrogen peroxide treatment of cultured cells, and the first to suggest that they contribute to tendon cell mineralization.

The involvement of adenosine in HO has been suggested in several studies focused on genetic disorders associated with HO. However, its theoretical mechanisms remain incompletely understood, as several conflicting explanations exist. In the present study, we tested our hypothesis by examining the effects of adenosine at relatively high concentrations in the osteogenic medium because lower concentrations did not show clear results. It is unknown why such a high dose of adenosine was required to see a significant increase in calcification. Additional pathways exerted by other ex-ATP metabolites may be one reason [27]. However, rather than examining multiple metabolites, which might compromise the clarity of the experimental design, we focused on adenosine as the sole factor. The involvement of other secretory factors, such as growth factors and cytokines, is also possible, as many previous studies have shown that oxidative stress often induces the production of various growth factors in different culture conditions and cell types. For example, negative and positive feedback loops between TGF-beta and oxidative stress have been reported in several systems, particularly in the context of tissue fibrosis in human disorders [28]. Also, mutual interactions between the BMP pathway and oxidative stress have been reported in lung fibroblasts and myoblasts [29,30]. Since BMPs and TGF-beta are important inductive factors in osteogenesis, increased or decreased secretion of these factors following hydrogen peroxide treatment may have influenced the results of our osteogenic assays. However, rather than investigating such possibilities beyond the scope of this study, we considered the possible reversal effects of adenosine on mineralization between the inside and outside of cells.

If the hydrolysis of ex-ATP following hydrogen peroxide treatment increases extracellular adenosine, it may support mineralization by binding to A2 receptors [13,27]. On the other hand, the immediate internalization of adenosine may occur through the transmembrane adenosine transporter ENT1. Since internalized adenosine may increase intracellular AMP levels, thereby activating AMPK, it may inhibit cell activity and proliferation [13,27]. This notion was supported by subsequent experiments in which the adenosine transporter inhibitor dipyridamole was added to osteogenic media supplemented with a low concentration (0.1 mM) of hydrogen peroxide. In this experiment, low-dose hydrogen peroxide was added with the expectation that it would induce the production of secretory factors in the extracellular milieu, including nucleotide metabolites at suboptimal levels. Subsequently, we observed that tendon cell mineralization was enhanced by the addition of the Ent 1 inhibitor dipyridamole to the culture. However, because we did not test whether Ent1 directly internalizes extracellular adenosine into the cytosol, it is necessary to examine the extracellular adenosine levels in the conditioned medium used in the experiment after the addition of low-dose hydrogen peroxide and dipyridamole. The limitations of our study also include the lack of examination of potential contributions from other secretory factors, including the growth factors described above. Future studies should examine such contributions and test the hypothesis that Ent1 takes up adenosine under these conditions or that specific antagonists can block the effects of hydrogen peroxide to strengthen mineralization in culture. It is also important to verify whether the incorporated AMP enhances AMPK activity and whether this enhancement suppresses osteogenesis [27]. Furthermore, the transmethylation pathway mediated by S-adenosylhomocysteine via internalized adenosine [27] could also be examined. In addition, further investigation into the mechanism of direct osteogenic differentiation via receptors for extracellular ATP, ADP, AMP, and adenosine is required in a more specialized in vivo environment.

Our study has clinical relevance, as it partially elucidated the mechanistic basis of ectopic ossification across various pathological settings, particularly in dense fibrous connective tissues such as tendons and ligaments, as well as in the arterial wall. Specifically, the findings may lead to a better understanding of the pathogenesis of spinal ligament ossification, which may cause serious spinal myelopathy. Other examples of ossification are HO after ligament injury, which can lead to joint contracture, and traumatic “myositis ossificans”, which can lead to a severe joint contracture. Valvular calcification of the heart is also an ossification originating from similar cell types. Ossification in an in vivo environment does not always reflect the results obtained in cell culture systems. However, findings from simplified culture conditions reflect at least some of the in vivo mechanisms of ossification and can inform the development of effective therapeutic or preventive methods in the future.

In summary, low concentrations of hydrogen peroxide enhanced calcification during the osteogenic induction of tendon cells. Transferred conditioned medium reproduced this effect without significant cell death. In the conditioned medium, increased levels of ex-ATP metabolites were confirmed by mass spectrometry approximately 12 h after hydrogen peroxide treatment.

Since our results revealed a novel regulatory mechanism in soft tissue calcification that implies therapeutic targets for heterotopic ossification, future studies will examine therapeutic effects in an in vivo model of tendon ossification. Related molecular mechanisms of ATP excretion after oxidative stress, as well as the impact of oxidative stress on calcification, could potentially be investigated [29].

## 5. Conclusions

Our study experimentally supports the involvement of ex-ATP metabolites in the molecular mechanisms underlying hydrogen peroxide-induced enhancement of tendon cell calcification during osteogenic induction. Further investigations are required to verify the various related mechanisms.

## Figures and Tables

**Table 1:** Primer sequences used for quantitative RT-PCR (mouse).

Gene Symbol	Forward Primers	Reverse Primers
*Osx*	5′-AGCGACCACTTGAGCAAACATC-3′	5′-CGGCTGATTGGCTTCTTCTTCC-3′
*Ocn*	5′-AGCTTAACCCTGCTTGTGCG-3′	5′-GGAGGATCAAGTCCCGGAGA-3′
*Alp*	5′-GCTATCTGCCTTGCCTGTATCTG-3′	5′-AGGTGCTTTGGGAATCTGTGC-3′
*Col1a1*	5′-CTGACTGGAAGAGCGGAGAG-3′	5′-GCACAGACGGCTGAGTAGG-3′
*Enpp1*	5′-GGACGCTATGATTCCT-3′	5′-GCTGGTGAGCACAATG-3′
*Mgp*	5′-GGCGAGCTAAAGCCCAAAAG-3′	5′-GTAGTCATCGCAGGCCTCTC-3′
*Ent1*	5′-GGAGAGGAGCCAAAAG-3′	5′-ACAGCAGGGAACAACC-3′
*Adra2a*	5′-CCTCGGTTTCCCCCAGC-3′	5′-CTGGCTCCACTCAGCTTCAG-3′
*Adra2b*	5′-CGTCCCGCTCAGGTATAAAGG-3′	5′-CAATGCCAAAGGCAAGGACC-3′
*Gapdh*	5′-AGAAGGTGGTGAAGCAGGCATC-3′	5′-CGAAGGTGGAAGAGTGGGAGTTG-3′

## Data Availability

The datasets generated and or analyzed during the current study are available from the corresponding author upon reasonable request.

## Supplementary Materials

The following supporting information can be downloaded at: https://www.mdpi.com/article/10.3390/cimb48030244/s1.

## Abbreviations

The following abbreviations are used in this manuscript:

HO	Heterotopic ossification
ATP	Adenosine triphosphate
ADP	Adenosine diphosphate
AMP	Adenosine monophosphate
ROS	Reactive oxygen species
P2X	Purinergic receptor 2X
P2Y	Purinergic receptor 2Y
P2	Purinergic receptor
A1	Adenosine A1 receptor
A3	Adenosine A3 receptor
Pi	Inorganic phosphate
PPi	Pyrophosphate
ABCC6	ATP-binding cassette subfamily C member 6
GACI	Generalized arterial calcification infancy
ENPP1	Ectonucleotide pyrophosphatase/phosphodiesterase 1
NT5E	Nucleotidase ecto
ENT1	Equilibrative nucleoside transporter 1
SLC29A1	Solute carrier family 29 member A1
FDL	Flexor digitorum longus
SV40	Simian virus 40
TCs	Tendon cells
Osx	Osterix
Ocn	Osteocalcin
Alp	Tissue nonspecific alkaline phosphatase
Mgp	Matrix Gla-protein
Gla	Gamma-carboxyglutamic
RT	Reverse transcription
PCR	Polymerase chain reaction
ANOVA	Analysis of variance
CM	Conditioned medium
OGM	Osteogenic culture growth medium
LC	Liquid chromatography
MS	Mass spectrometry
HPLC	High-performance liquid chromatography
MRM	Multiple reaction monitoring
SD	Standard deviation
