# Supplementary material for: Extracellular Adenosine Contributes to the Hydrogen Peroxide-Induced Calcification of Cultured Tendon Cells
Source: Curr Issues Mol Biol. 2026 Feb 26;48(3):244. doi: 10.3390/cimb48030244 (PMC13025900; doi:10.3390/cimb48030244)
Supplement: Supplementary file 1 [file cimb-48-00244-s001.zip › cimb-4096521-supplementary.pdf]

Figure S1

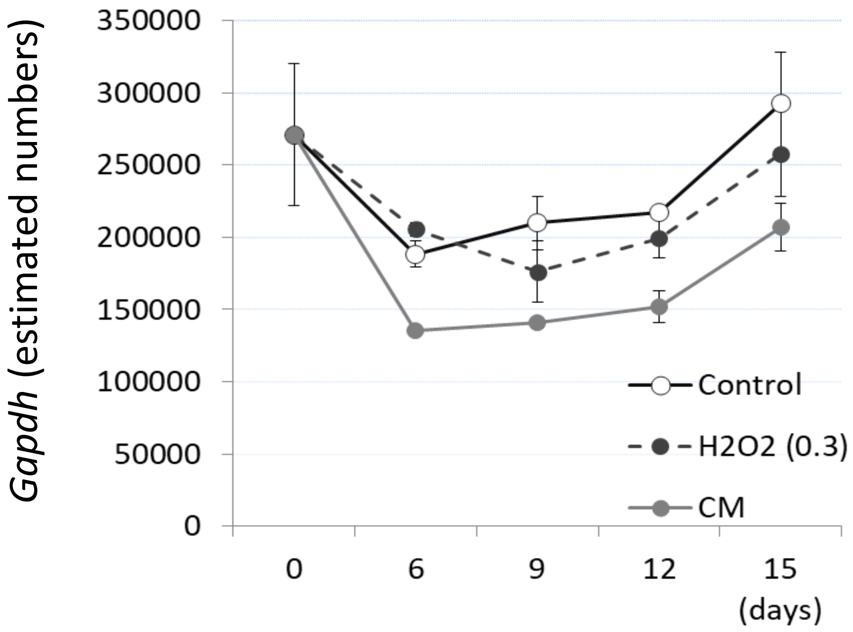

Gapdh stability during the course of osteogenic assay in TT-D6 cells

Semiquantitative RT-PCR was performed at the indicated time points using Gapdh-specific primers in TT-D6 cells cultured for the osteogenic induction. The vertical axis presents the levels of expression estimated as molecular numbers in the template cDNA.

Figure S2

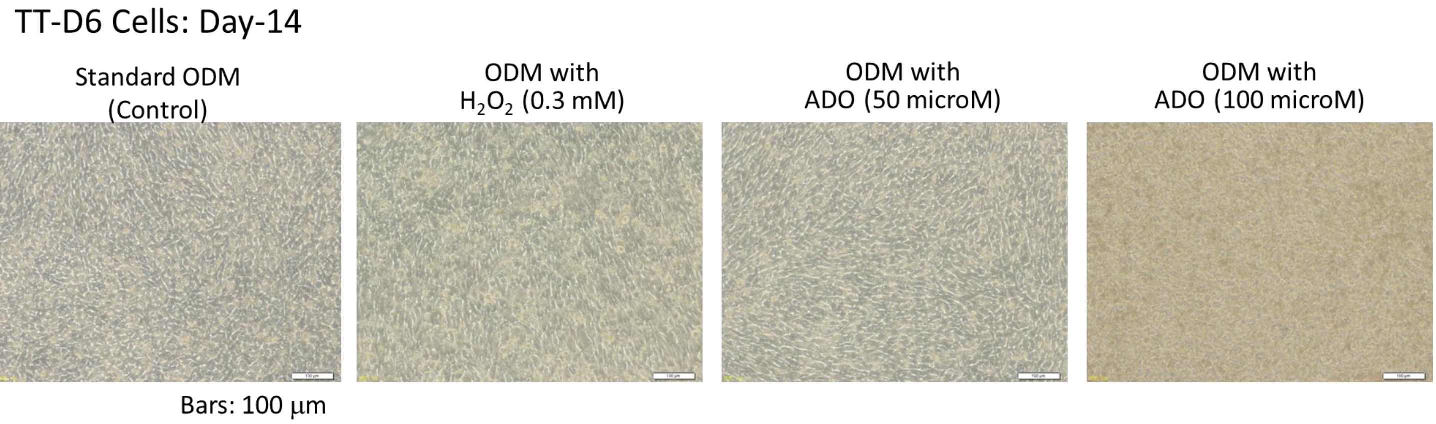

Examples of microscopic images of cultured TT-D6 cells after 14 days of osteogenic assay.

Osteogenic induction of the TT-D6 cells was done with extrinsic adenosine treatment (50 or 100 micromolar) or with hydrogen peroxide at 0.3 mM. Microscopic images were acquired prior to alizarin red staining. ODM: osteogenic differentiation medium

Figure S3

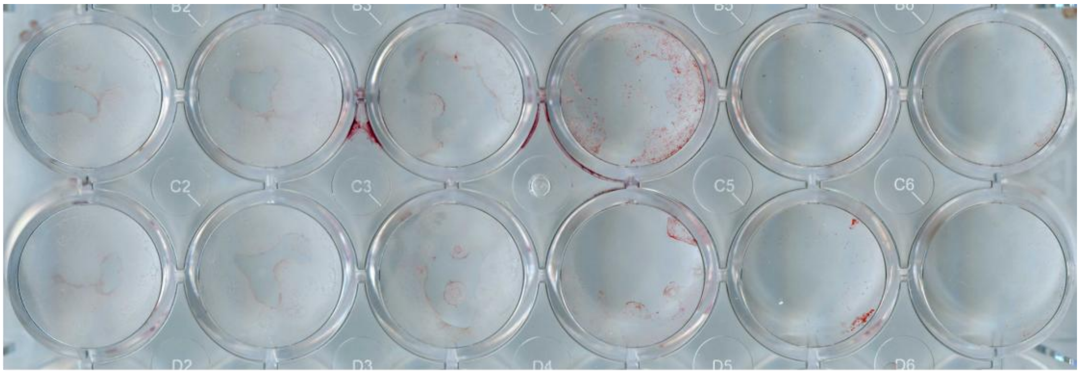

H_2O_2 (mM)	-	0.1	0.2	0.3	0.4	0.5

Alizarin Red staining at 12 days of the TT-D6 cell osteogenic assay with different concentrations of H_2O_2 as a pilot study.

Figure S4

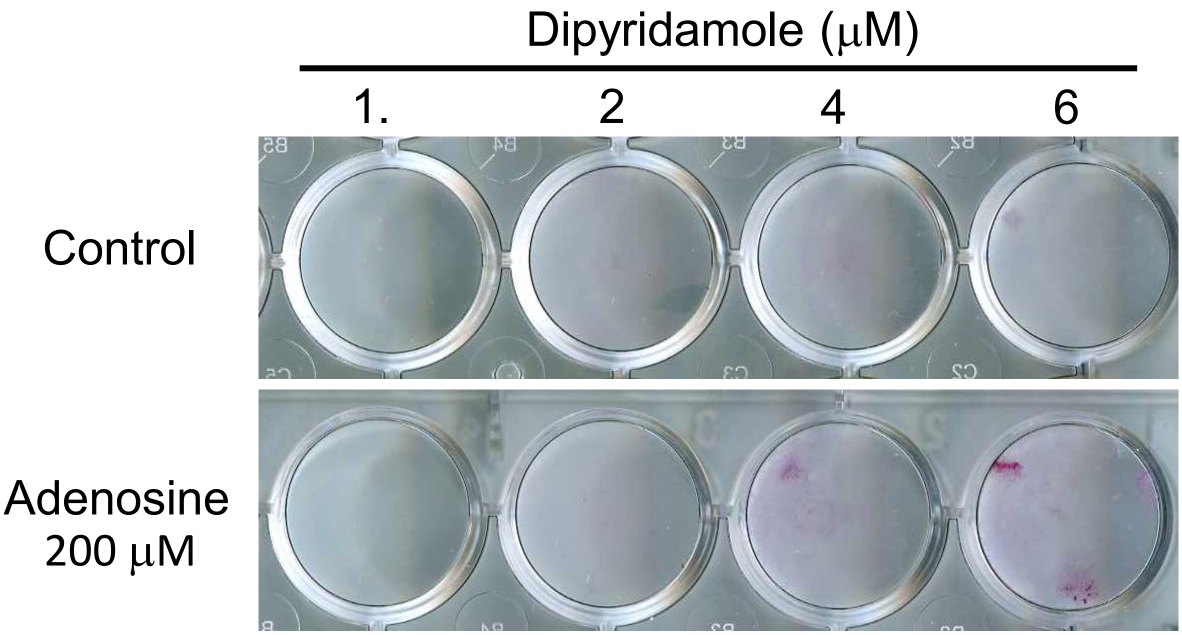

An inhibitor of the adenosine transporter Ent1, dipyridamole, enhanced the calcification of TT-D6 cells subjected to the osteogenic culture.

Osteogenic induction of TT-D6 cells was performed with extrinsic adenosine treatment (200 micromolar in this experiment to detect differences), with or without dipyridamole at four concentrations (1, 2, 4, 6 micromolar). 1.0 dipyridamole was used as the control because a lower concentration appeared to slightly affect calcification. The calcifications were evaluated by alizarin red staining.

Figure S5

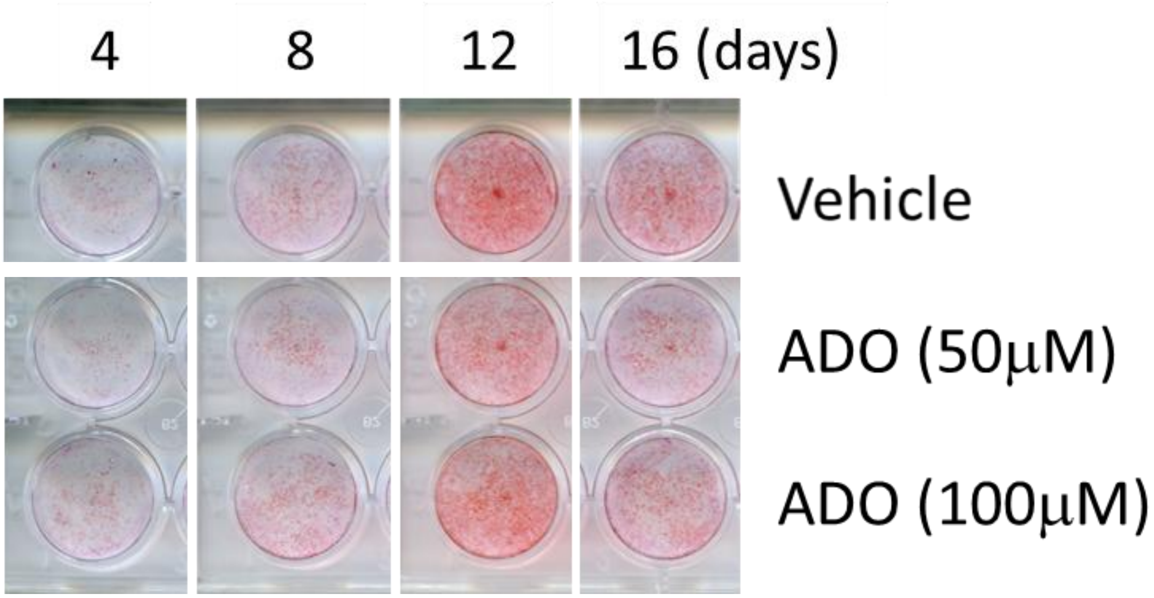

High-dose adenosine enhanced the calcification of the primary FDL tendon cells subjected to the osteogenic culture.

Osteogenic induction of the FDL tendon cells was done with extrinsic adenosine treatment (50 and 100 micromolar). The calcifications were evaluated by alizarin red staining at days 4, 8, 12, and 16 of the culture experiment.

Figure S6

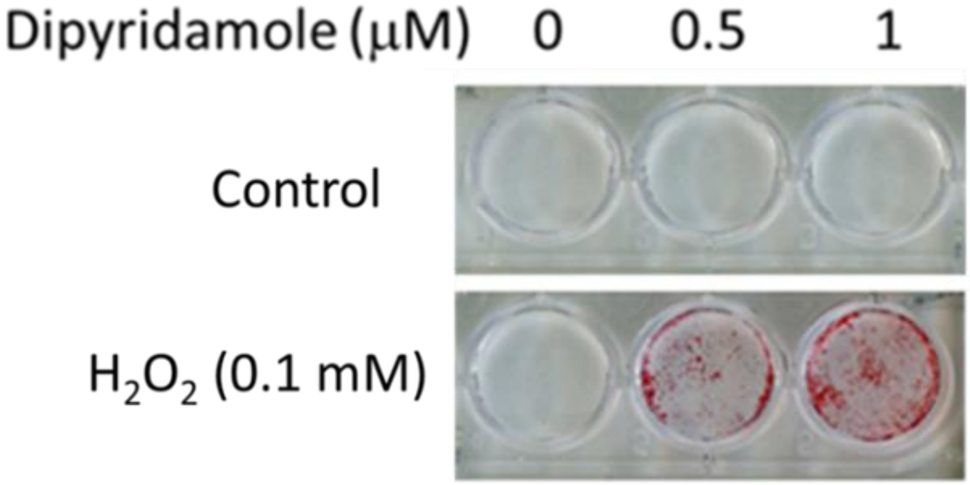

An inhibitor of the adenosine transporter Ent1, dipyridamole, enhanced the calcification of the FDL tendon cells subjected to the osteogenic culture.

Osteogenic induction of the FDL tendon cells was done with low-dose hydrogen peroxide treatment (0.1 millimolar) with different concentrations of dipyridamole (0, 0.5, or 1.0 micromolar). The calcifications were evaluated by alizarin red staining at day 16 of the culture experiment.

Table S1. MRM parameter for nucleotide methods

Metabolite name	Precusor Ion (m/z)	Product Ion (m/z)	Fragment voltage (V)	Collision energy (V)	Cell accelerator (V)	Retention time (min)	Retention time window (min)	Polarity
AMP	348	136	110	20	5	3.72	2.0	Positive
ADP	428	136	120	30	5	4.36	2.0	Positive
ATP	508	136	140	38	5	4.84	2.0	Positive
Methionine sulfone (I.S.)	182	56	80	20	5	2.16	2.0	Positive

Table S2. MRM parameter for nucleoside methods

Metabolite name	Precusor Ion (m/z)	Product Ion (m/z)	Fragment voltage (V)	Collision energy (V)	Cell accelerator (V)	Retention time (min)	Retention time window (min)	Polarity
Deoxyadenosine	252	136	100	14	5	1.98	1.5	Positive
Deoxyguanosine	268	152	80	10	5	3.96	1.5	Positive
Deoxycytidine	228	112	170	8	5	3.57	1.5	Positive
Deoxyuridine	229	113	80	4	5	1.73	1.5	Positive
Deoxyinosine	253	137	60	6	5	3.06	1.5	Positive
Adenosine	268	136	100	20	5	2.54	1.5	Positive
Guanosine	284	152	80	14	5	4.33	1.5	Positive
Cytidine	244	112	180	10	5	3.91	1.5	Positive
Uridine	245	113	80	8	5	2.50	1.5	Positive
Inosine	269	137	80	8	5	3.58	1.5	Positive
Thymidine	243	127	70	10	5	1.51	1.5	Positive
Xanthosine	285	153	80	6	5	4.62	1.5	Positive
Adenine	136	119	80	24	5	2.32	1.5	Positive
Guanine	152	135	100	20	5	3.83	1.5	Positive
Cytosine	112	95	130	20	5	3.33	1.5	Positive
Uracil	113	70	100	16	5	1.33	1.0	Positive
Thymine	127	110	100	16	5	1.20	1.0	Positive
Hypoxanthine	137	55	120	36	5	2.71	1.5	Positive
Xanthine	153	110	120	18	5	2.77	1.5	Positive
Uric acid	167	124	120	14	5	4.81	2.0	Negative
5-Methylcytidine	258	126	180	12	5	3.70	1.5	Positive
5-Methyldeoxycytidine	242	126	180	8	5	3.38	1.5	Positive
5-Methylcytosine	126	109	140	22	5	3.16	1.5	Positive
Methionine sulfone (I.S.)	182	56	80	20	5	5.31	3.0	Positive
